# Papillomatous Ovarian Growths

**Published:** 1891-12-26

**Authors:** 


* *Therap. Gaz.*, Sept. 15, 1890.

### SURGERY.

### PAPILLOMATOUS OVARIAN GROWTHS.

We have lately had under observation a case of ascites in a young woman, in which on opening the abdomen large papillomatous growths were found in the pelvis. Some were as large as an orange, others very much smaller. They all exactly resembled a cauliflower in appearance. The larger ones were pedunculated, the smaller sessile. They grew from the broad ligament and posterior surface of the uterus, and were associated with numerous cysts the size of a hen's egg and smaller, each containing the same kind of papillary growths as those apparently extra-cystic. The ovaries were not discovered at the operation, and no sign of a large cyst existed. The intestines were considerably matted together at the back of the abdomen. The fluid in the peritoneal cavity was distinctly blood-stained, as had been the case on a previous tapping. Some obscure masses could be felt in the pelvis before operation, but the ascites was thought to be due to chronic tubercular peritonitis.

What is the origin of the papillomatous growths in these cases? In by far the majority an ovarian papillomatous growth is intra-cystic. Cyst arising in connection with the remains of the Wolffian body, whether in the parovarium or hilum of the ovary or elsewhere in the broad ligament, may contain papillary intra-cystic growths. In a few rare cases the same kind of growth is found on the surface of the ovary. Doran figures such a case, and another beautiful illustration of such a condition is to be found in the American system of Gynæcology; but these cases are very rare, and to make sure of their being of this nature, one must be able to demonstrate their origin from the ovary, and not merely from the broad ligament. That we do not find these papillomatous growths in cysts does not disprove their intra-cystic origin. Sometimes the cyst ruptures, and so the papillomatous growths are let loose as it were into the peritoneal cavity, and this we believe was their origin in this case, looking at the fact that they were associated with cysts containing the same kind of growth. The larger cysts containing them are occasionally quite thin walled and easily ruptured. When growing on the free surface of the ovary they are not as a rule associated with cysts. Both true ovarian papilloma and free papillomatous growths that were once intra-cystic, are very likely to cause the ascitic fluid to be tinged with blood. This is so also in ascites due to malignant disease in the abdomen.

They are not malignant growths in the ordinary sense of the term, but when once they get free in the peritoneal cavity they disseminate very rapidly, and in this way run the course of malignant growths. Sometimes they protrude through the cyst wall, and thus infect the peritoneum, at others through rupture of the cyst. But as Mr. Lawson Tait points out, now and then, when it has not been possible to remove all the growth, the clinical history of the case has seemed to show that they have been absorbed, or, at any rate, they gave no further trouble. But this is not always so; sometimes, on the other hand, they disseminate very rapidly in the peritoneal cavity.

Clinically we cannot tell whether we have papillary growths in an ovarian cyst or not; and looking at the fact that they may be present, and may grow through the cyst wall (leaving out of consideration the less likely occurrence of rupture), the possibility of their existence should always be remembered in deciding for or against very early removal. These papillomatous cysts are often cysts in the broad ligament, without any pedicle, pushing the uterus over to the other side.

Microscopically they are composed of a stem of interlacing fine connective tissue with some round and branching cells scattered about in it, and covered with columnar epithelium. In some of the terminal processes we have found spindle and round-celled tissue like embryonic tissue, but taking the growth as a whole it is not sarcomatous, and there is no in-growth of surface epithelium.

